# *PRDM16* Represses the Pig White Lipogenesis through Promoting Lipolysis Activity

**DOI:** 10.1155/2019/1969413

**Published:** 2019-06-13

**Authors:** Ting Gu, Guli Xu, Chengfeng Jiang, Lianjie Hou, Zhenfang Wu, Chong Wang

**Affiliations:** ^1^National Engineering Research Center for Breeding Swine Industry, Guangdong Provincial Key Lab of Agro-Animal Genomics and Molecular Breeding, College of Animal Science, South China Agricultural University, Guangzhou 510642, China; ^2^Wens Foodstuff Group Co., Ltd., Yunfu, China

## Abstract

The positive regulatory domain containing 16 (*PRDM16*) gene is a dominant transcriptional regulator that favors the “browning” of white adipocytes in rodents. Since the “browning” of white fat is important in pig in terms of producing heat fighting against cold environment, avoiding obesity, and improving meat quality, understanding the critical role that* PRDM16* gene played in pig adipose “browning” and energy metabolism is of great significance. However, the constitution of pig fat differs a lot from rodents and human as they do not have brown adipose tissue (BAT) even in the newborn piglets. In this study, we isolated porcine primary preadipocytes and investigated the function of* PRDM16* during preadipocytes differentiation. Our results showed that overexpression of the PR domain of* PRDM16* repressed the differentiation of porcine preadipocytes, indicated by oil red O staining and the deposition of the triglyceride. Overexpression of the PR domain significantly increased the level of lipolysis and mitochondrial oxidative capacity detected by Western blotting during differentiation. Furthermore, we purified the protein coded by the PR domain and demonstrated that this protein has the H3K9me1 methyltransferase activity. In conclusion, the PR domain of the porcine* PRDM16* gene repressed the mature of the porcine preadipocytes by promoting its oxidative activity.

## 1. Introduction

Adipose tissue is mainly divided into two types: white adipose tissue (WAT) and brown adipose tissue (BAT), which are mainly composed of white and brown adipocytes, respectively [1]. The white adipocytes can be turned into browning-like adipocytes under cold challenge, namely, beige adipocytes. The brown and beige adipocytes dissipate energy and produce heat, which is important in fighting with cold stress and obesity, while the WAT is not only an energy storage but also an endocrine secreting organ that plays important roles in energy balance [2]. This is especially important in swine production industry as the temperature variation during early postnatal period is strongly correlated with the high mortality of the preweaning piglets [3].

Previous studies showed that although brown and beige cells shared a lot of common characters, both of them were abundant of mitochondria and are UCP1+ cells, and they derived from different precursors, as both white and beige adipocytes derived from the committed white adipocyte precursors, while the brown adipocytes derive form the same precursors with the muscle cells [4]. As mentioned above, in response to chronic cold stimulation, the white adipose can convert into beige fat cells, which improves the number of mitochondria and the rate of lipolysis to generate more heat to fight against the cold stress, while the opposite transition can happen after warm adaption [5]. The development of the white adipocyte is delicate controlled by several regulators, such as peroxisome proliferator-activated receptor-gamma (PPAR-*γ*), C/EBP*α*, C/EBP*β*, and epigenetic modification regulators including HDAC9 and SIRT1 [4, 6]. The PR domain containing 16 (*PRDM16*), also called MDS1/EVI1-like gene 1(*MEL1* gene), contains an N-terminal PR domain, which is the key functional domain of this gene. Baizabal et al. found that the PR domain is the vital component of the gene in regulating upper layer neuron migration by comparing reintroducing* PRDM16* and the* PRDM16* deleted the PR domain (△PRDM16) into the cKO radial glial cells [7]. Studies in human and rodents showed that* PRDM16 *played crucial roles in regulating the development of fat tissue. In human, the* PRDM16* polymorphisms are associated with obesity as the A allele is dominant in the obese group [8]. The study in adipocyte-specific knockout mice also demonstrated that* PRDM16* mainly affected the “browning” of the white adipocyte when stimulated with cold stress, and knockout of this gene will lead a switch from subcutaneous to visceral fat [9].* PRDM16* regulated beige adipocyte development by suppressing the expression of white fat-selective genes, which recruited the histone methyltransferase Ehmt1 in young mice [10]. Overexpression of* PRDM16* in the fibroblast cell model C2C12 also leads to repression of myogenic genes and adipogenic genes in both proliferation and differentiation stages, indicating* PRDM16* switched the cell fate into brown adipocyte [11]. Furthermore,* PRDM16* can be regulated by other posttranscriptional factors, such as miRNAs, sumoylation, and histone deacetylase 3(HDAC3) in the process of browning and thermogenesis in white adipocyte [12–14]. The PR domain of* PRDM16* may work by its H3K9me1 transferase activity, as this domain of the* PRDM* family shares high homology with the SET domain, which possesses the activity of the histone methyltransferase [15]. This is demonstrated by the study in mouse embryonic fibroblasts that* PRDM16 *functioned as the lysine methyltransferase (KMT) directing cytoplasmic H3K9me1 methylation, which methylated H3K9me1 and then can be converted into H3K9me3 in nucleus to reinforce the heterochromatin [16].

The composition of pig adipocyte is quite different from rodents and human, and pig lacks the brown adipocyte while the key regulator* UCP1* was disrupted about 20 million years ago [17, 18]. Recently, studies found that the cold resistant pig breeds, such as Min and Tibetan pigs, can fight against the cold environment pressure in a nonshivering dependent manner, by expressing* UCP3* gene to induce the browning of white adipocyte as an evolution mechanism [19]. Furthermore, since pig lacks the functional* UCP1* gene, Zhao's group knocked the functional mice* UCP1* into pig genome using CRISPR-Cas9 system, and found that the thermoregulation ability was improved; however, the white adipocytes were not “browned” [20], so the mechanism of “browning” or metabolism mechanism of pig white adipocytes is still unclear. As a result, the aim of this study was to investigate the role of* PRDM16* in the differentiation of pig white adipocyte and energy metabolism, which may provide some new insight in the way of fighting with obesity and improving the living number of newborn piglets.

## 2. Materials and Methods

### 2.1. Animals

All animals used in this study were reared and euthanized with the approval of the College of Animal Science, South China Agricultural University. We followed the methods of Hou et al. 2017 that we published previously [21]. All experiments were conducted following “the instructive notions with respect to caring for laboratory animals” issued by the Ministry of Science and Technology of the People's Republic of China.

### 2.2. pcDNA-3.1-PR Vector Construction

For constructing the ectopic expression vector, porcine PR fragment was amplified by the primer pair PR-2 with NheI- BamH sites in the end and the PCR product was digested and cloned into the pcDNA3.1 vector. PRDM16-2F: 5′-* CTA*GCTAGCACTCCCAAGGAAGGCTCG-3′; PRDM16-2R: 5′-* CGC*GGATCCCTTTGTGGCGTCGCAGGTC-3′. The DNA base pairs in italic are the protective bases and the base pairs in rectangle are the restriction enzyme recognition site sequences. The PCR amplification condition is as follows: 94°C 5 min; 94°C, 30 s, 63°C, 30 s, 72°C, 40 s, repeated 35 cycles; 72°C, 10 min.

### 2.3. Porcine Preadipocyte Isolation and Culture

Porcine preadipocytes were isolated from subcutaneous adipose tissue of 1-day-old male Landrace pigs' back according to previous published protocols with modifications [9, 21–23]. Briefly, subcutaneous adipose tissue was isolated and finely minced after removing all visible connective tissues and then digested for 40 min at 37°C in isolation buffer (125 mM NaCl, 5 mM KCl, 1.3 mM CaCl2, 5 mM glucose, 100 mM HEPES, 4% BSA, 1.5 mg/ml Collagenase B). Digested tissue was filtered through a 100 *μ*M cell strainer to remove large pieces, and the flow-through was centrifuged for 10 min at 500 ×* g* to collect stromal-vascular fraction (SVF) cells in the pellet. The SVF cells were suspended in growth medium-DMEM/F12 (GIBCO, Grand Island, NY, USA) containing 15% fetal bovine serum (FBS, GIBCO), 100,000 units/L of penicillin sodium, and 100 mg/L of streptomycin sulfate (GIBCO) and then plated onto 10 cm tissue culture dishes. For PR domain overexpression, preadipocytes with 60% confluent were incubated with pcDNA-3.1-PR or pcDNA-3.1 backbone plasmid for 6 h in growth medium. Then the medium was replaced with new growth medium and cells were maintained in growth medium for an additional 12 h before adipogenic differentiation induction. Preadipocytes were induced to differentiation by the induction medium containing 10% FBS, 0.5 mM isobutylmethylxanthine, 0.25 *μ*M dexamethasone, 1 *μ*g/mL insulin, and 1 *μ*M rosiglitazone for 48 h. Two days later, cells were switched to a different medium containing 10% FBS and 1 *μ*g/mL insulin. Differentiated cells were collected for gene expression detection, and culture media were used to conduct triglyceride assays. All chemicals for cell culture were purchased from Sigma-Aldrich (St. Louis, Missouri, United States).

### 2.4. Triglyceride Measurement

Porcine preadipocytes were differentiated for 5 or 8 days and homogenized in ice-cold phosphate-buffered saline (PBS). After centrifugation (1000* g*, 10 min), the supernatants were collected for triglyceride analysis following the manufacturer's instructions (Applygen, Beijing).

### 2.5. Oil Red O Staining

Differentiated cells were washed three times with PBS, followed by fixation with 4% paraformaldehyde in phosphate buffer for 35 min at room temperature. After fixation, the cells were washed again with PBS twice and stained with fleshly diluted Oil red O, which was made up of 2 proportions of PBS and 3 proportions of stock solution for 10 min. The cells were then washed with PBS for 3 times (gently to avoid cell floating). The pictures were taken under inverted microscope.

### 2.6. Western Blotting

The porcine preadipocytes on different differentiation stages were collected and the protein was extracted according to the instruction of RIPA lysis buffer (P0013B, Beyotime). The concentration of protein was detected by BCA protein essay kit (23225, Thermo Fisher) and then adjusted to 1 *μ*g/*μ*l for SDS-PAGE gel running. The proteins were subjected to Western blotting analysis with the following antibodies: anti-PPAR*γ* (2435S, 1:1000, CST), anti-C/EBP*α* (8178T, 1:1000, CST), anti-AP2 (D120618, 1:500, Sangon Biotech), anti-COX3 (D160197, 1:500, Sangon Biotech), anti-ELOVL (D122292, 1:500, Sangon Biotech), anti-ACOX1 (D121471, 1:500, Sangon Biotech), anti-HSL (18381T, 1:1000, CST), anti-UCP3 (K002819P-50ul, 1:1000, Solarbio), anti-*β*-actin (AP0060, 1:5000, Bioword). The blots were developed using HRP-conjugated secondary antibodies and ECL system. The relative expression levels were calculated by software Image J in the form of the band density relative to *β*-actin.

### 2.7. pET-28a-PR Vector Construction and Protein Purification

The PR domain was cloned into the pET-28a vector which contains a his-tag. The fragment was amplified by the primer pair PR-2 with NheI(GCTAGC) - BamHIsites (GGATCC) and the stop codon (CTA) in the end and the PCR product was digested and cloned into the pET-28a vector. PRDM16-2F: 5′-TGGCTAGCCACTCCCAAGGAAGGCTCG -3′; RDM16-2R: 5′-TTGGGATCC*CTA*GAAGGTGGGCTCCTCGTCC-3′. The PCR amplification condition is as follows: 94°C 5 min; 94°C, 30 s, 63°C, 30 s, 72°C, 40 s, repeated 35 cycles; 72°C, 10 min. After transfection and sequencing for the insertion sequence, the vector was expressed in the* E. coli* BL21 (DE3) and the combined protein was purified using the Ni-NTC columns.

### 2.8. Histone Methyltransferase Activity Measurement

10 *μ*l of the total histone (Sigma-aldrich) was incubated with the purified PR-his combinant protein or his protein as the negative control in 30°Cfor 1 h. Western blotting with H3K9me1 antibody was used to detect the methyltransferase activity of the PR protein.

### 2.9. Statistical Analysis

The levels of triglyceride deposition and gene relative expression were presented as mean ± standard error of the mean (SEM). Student's* t*-test was carried out using the R (download website: https://www.r-project.org/).

## 3. Results

### 3.1. Overexpression of the PR Domain of the* PRDM16* Gene Repressed the White Adipogenesis

**Figure 1:**
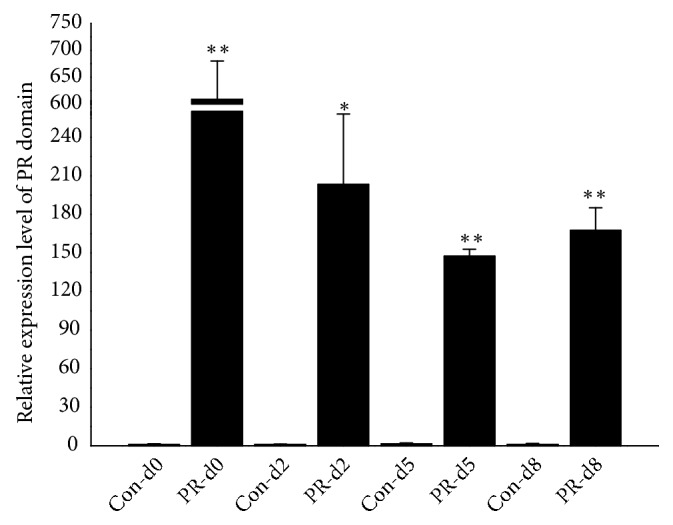
qPCR analysis of the mRNA level of PR domain in adipocytes transfected pcDNA3.1-PR or pcDNA3.1. The Con-dn in X-axis showing samples transfected pcDNA3.1 in different time points from day 0 to day 8 and the PR-dn showing samples transfected pcDNA3.1-PR. ^*∗∗*^ represented the *P* value <0.01 and ^*∗*^ represented the *P* value <0.01.

**Figure 2:**
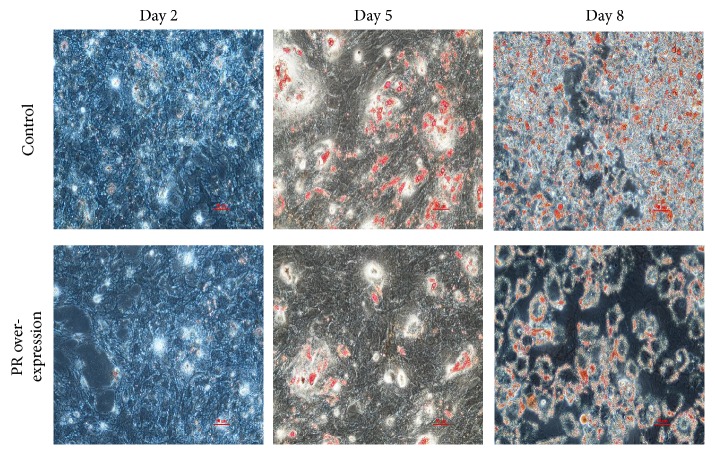
The oil red staining of adipocytes transfected with negative control (pcDNA3.1 basic) in the upper panel and pcDNA3.1-PR vector in the lower panel.

We isolated the porcine preadipocytes from subcutaneous adipose tissue of 1-day-old male Landrace pig and the pcDNA3.1-PR vector or the pcDNA3.1 vector was transfected when the cells reached the 60% confluence. After being incubated with the vector for 6 h, the cells were cultured in new growth medium and maintained for an additional 12 h before inducing for differentiation and the adipocytes in different time stages were collected for further molecular analysis. qPCR results showed that the PR domain was successfully transfected into porcine preadipocytes and expressed significantly higher than the blank vector (Figure 1). As shown in Figure 2, overexpression of the PR domain repressed the white adipogenesis, indicated by the lipid droplets stained by oil red O in mature adipocytes (day 8).

### 3.2. Overexpression of the PR Domain Repressed the Triglyceride Deposition

**Figure 3:**
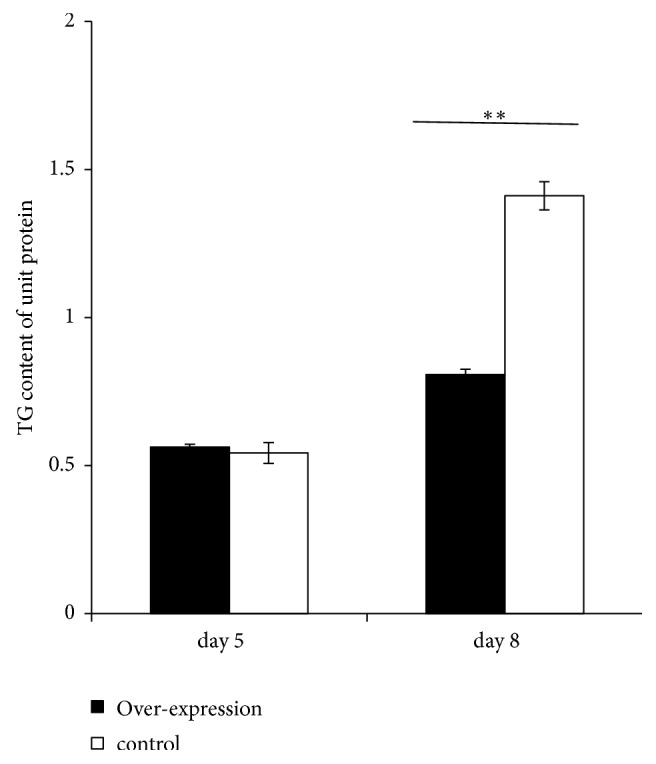
Effect of PR domain on the triglyceride deposition in differentiated adipocytes. ^*∗∗*^ represented the *P* value <0.01.

To test the effect of the PR domain on triglyceride deposition, we collected the differentiated mature adipocytes and homogenized in ice-cold phosphate-buffered saline (PBS). After centrifugation (1000* g*, 10 min), the supernatants were collected for triglyceride analysis. The results showed that the PR domain overexpression significantly repressed triglyceride deposition on the differentiation day 8 compared to the control, while on the differentiation day 5, no significant repression effect was observed (Figure 3).

### 3.3. The PR Domain of* PRDM16 *Repressed the White Adipogenesis by Promoting Its Oxidative Activity

**Figure 4:**
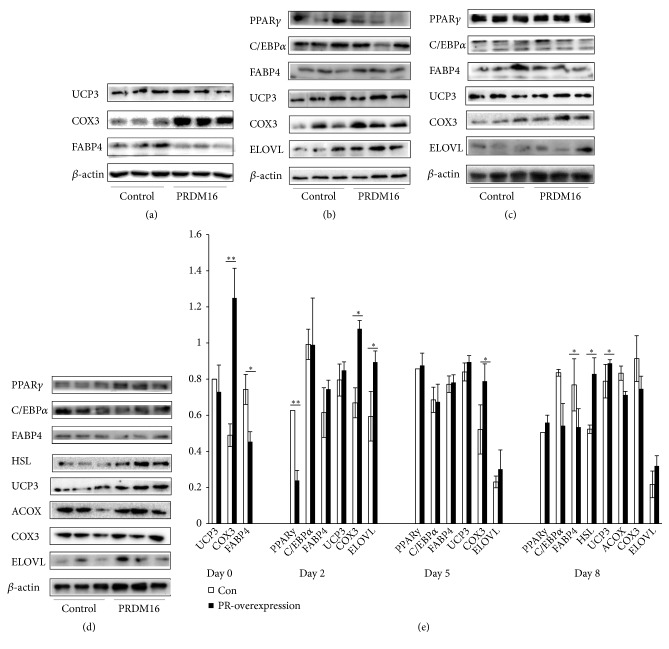
Western blot and densitometry analysis for protein levels on the general adipocyte markers and genes related to fatty acid oxidation and lipolysis in pig preadipocytes after transfecting the PR domain expressing vector or control vector. (a) On differentiation day 0; (b) on differentiation day 2; (c) on differentiation day 5; (d) on differentiation day 8. (e) Densitometry showing expression level of each gene in (a) to (d).

Since the* PRDM16* gene is a brown adipocyte determination factor and can stimulate mitochondrial biogenesis and uncouple cellular respiration in rodents, we further detected genes common to both white and brown fat lineage, such as peroxisome proliferator activated receptor gamma (PPAR*γ*), CCAAT enhancer binding protein alpha (C/EBP*α*), adipocyte protein 2 (AP2, also called FABP4, short for adipocyte fatty acid binding protein), and the brown fat-selective expressed genes, such as elongation of very long chain fatty acids (ELOVL) and mitochondrial electron transport gene Cytochrome C oxidase subunit III (COX3) by Western blot analysis in differentiated pig preadipocytes after transfecting the PR domain vector. The results demonstrated that the level of fatty acid metabolism was elevated by the PR domain, indicated by elevated expression of COX3 (on day 0: 1.25 ± 0.16 vs. 0.49 ± 0.08,* P* = 0.007; on day 2: 1.08 ± 0.05 vs. 0.67 ± 0.14,* P* = 0.02; on day 5: 0.79 ± 0.17 vs. 0.52 ± 0.06,* P *= 0.03 in Figures 4(a)–4(c), and 4(e)) and ELOVL (on day 2: 0.89 ± 0.06 vs. 0.59 ± 0.11,* P *= 0.03 in Figure 4(b)) in the adipocytes transfected the PR domain compared with the adipocytes transfected the control vector during differentiation.

Since pig does not have* UCP1* gene, we further check whether the thermogenic uncoupling process was activated in pig adipocytes by testing the protein expression level of pig specific browning gene* UCP3*. We also tested the lipolysis gene hormone-sensitive lipase (*HSL*), to make out whether the hydrolysis of fatty acids in cytoplasm was elevated in adipocytes. The results demonstrated that UCP3 was elevated (0.89 ± 0.02 vs. 0.79 ± 0.04,* P* = 0.04 in Figures 4(d) and 4(e)) in adipocyte transfected PR domain on differentiation day 8. The lipolysis gene HSL was also upregulated (0.83 ± 0.09 vs. 0.52 ± 0.09,* P* = 0.04 in Figures 4(d) and 4(e)) in the adipocytes transfected PR domain on differentiation day 8. These dates demonstrated that the PR domain was sufficient to promote the lipolysis and fatty acid oxidation.

### 3.4. Effect of PR Overexpression on Total Histone Methylation

**Figure 5:**
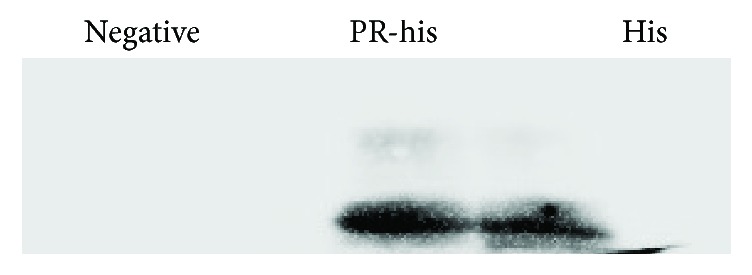
Western blot analysis of the H3K9me1 level using H3K9me1 antibody. Lane 1 was total histone (negative control); lane 2 was total histone with PR-his recombinant protein; lane 3 was total histone with his-tag.

We expressed the PR domain encoding protein by pET-28a vector, which carried a his-tag and can be further purified by the Ni-NTA column. As shown in Figure 5, PR-his recombinant protein methylated the total histone with a higher level compared to the his-tag protein using the H3K9me1 antibody for Western blot and indicated that the PR domain has the activity of H3K9me1 methyltransferase (Figure 5).

## 4. Discussion

A detailed understanding of the role that the* PRDM16* gene played in adipocyte differentiation and metabolism regulation would be beneficial not only for pharmaceutical development in fighting with obesity, but also for improving the living numbers of newborn farm animals who are weak in adapting the cold environment [24]. Pig is different with rodents and human in terms that even in the newborn piglets no brown fat tissue appears, which is confirmed by the loss of* UCP1* gene as we reported previously [21]. As a result, it is of greater importance to clarify the mechanism of adipocytes differentiation in pig.

In this study, we found that overexpression of the PR domain of* PRDM16 *repressed the adipogenesis in porcine preadipocytes and promoted fatty acid oxidation, indicated by the Western blot analysis in Figure 4 as alleviated AP2 (FABP4) and elevated COX3 were observed in the pig adipocytes overexpressing the PR domain on differentiation day 0. This result is consistent with the report in mice as lacking of AP2 would protect mice from diet-induced obesity [25]. On differentiation day 2, overexpression of the PR domain inhibited PPAR*γ*; however, there was no difference on differentiation day 5, and on differentiation day 8 the protein of PPAR*γ* was even more than the control group. This is reasonable as PPAR*γ* is a glitazone receptor, which was required for the survival of differentiated adipocytes [26]. In mice ES cells, the PPAR*γ* was expressed at high level immediately after differentiation was induced and it promoted lipogenesis depending on the gene dosage, and its expression decreased when differentiation continued [27]. Meanwhile, overexpressing the PR domain inhibited lipogenesis in porcine preadipocytes. On differentiation days 2 to 8, COX3 and ELOVL were significantly elevated. This is consistent with the results in C2C12 myoblasts, in which overexpression of the* PRDM16* gene by retroviruses would significantly elevate the expression level of ELOVL [28]. Furthermore, our result showed the higher fatty acid oxidation level in mitochondrial by overexpression of the PR domain was in coordination with the higher fatty acid lysis level in the cytoplasm, indicated by the higher expression of HSL on differentiation day 8. Moreover, the expression of UCP3 was also elevated in our study. This is consistent with the previous report as Lin et al. found that UCP3, instead of UCP1, was the thermogenic uncoupling protein in pig, which improved pig's ability to fight against cold [19].

The PR domain coding protein has the H3K9me1 methyltransferase activity, although the target genes regulated still need further study. Notably, the PR domain of* PRDM16* may not only possesses the H3K9me1 methyltransferase activity, as in the study in C2C12 showing that knockout of the PR domain not only affected the level of H3K9me1, but also H3ac, H3K4me3, and H3K27me3 on the promoter of the MyoD gene, although it may occurr through a complex network [11].

Based on our findings, we suggest a role for the PR domain of the PRDM16 gene in repressing the pig lipogenesis by promoting the lipolysis activity through H3K9me1 activity, although more investigations are still in need to clarify the mechanism behind.

## Data Availability

The data used to support the findings of this study are available from the corresponding author upon request.

## References

[1] Cannon B., Nedergaard J. (2008). Developmental biology: neither fat nor flesh. *Nature*.

[2] Jung S. M., Sanchez-Gurmaches J., Guertin D. A. (2018). Brown adipose tissue development and metabolism. *Handbook of Experimental Pharmacology*.

[3] He T., He L., Gao E. (2018). Fat deposition deficiency is critical for the high mortality of pre-weanling newborn piglets. *Journal of Animal Science and Biotechnology*.

[4] Cristancho A. G., Lazar M. A. (2011). Forming functional fat: a growing understanding of adipocyte differentiation. *Nature Reviews Molecular Cell Biology*.

[5] Rosenwald M., Perdikari A., Rülicke T., Wolfrum C. (2013). Bi-directional interconversion of brite and white adipocytes. *Nature Cell Biology*.

[6] Rosen E. D., MacDougald O. A. (2006). Adipocyte differentiation from the inside out. *Nature Reviews Molecular Cell Biology*.

[7] Baizabal J., Mistry M., García M. T. (2018). The epigenetic state of PRDM16-regulated enhancers in radial glia controls cortical neuron position. *Neuron*.

[8] AlAmrani A., AbdelKarim M., AlZoghaibi M. (2018). PRDM16 gene polymorphism is associated with obesity and blood lipids profiles in Saudi population. *Journal of Clinical Medicine*.

[9] Cohen P., Levy J. D., Zhang Y. (2014). Ablation of PRDM16 and beige adipose causes metabolic dysfunction and a subcutaneous to visceral fat switch. *Cell*.

[10] Harms M. J., Ishibashi J., Wang W. (2014). Prdm16 is required for the maintenance of brown adipocyte identity and function in adult mice. *Cell Metabolism*.

[11] Li X., Wang J., Jiang Z., Guo F., Soloway P. D., Zhao R. (2015). Role of PRDM16 and its PR domain in the epigenetic regulation of myogenic and adipogenic genes during transdifferentiation of C2C12 cells. *Gene*.

[12] Chen Q., Huang L., Pan D., Zhu L. J., Wang Y. X. (2018). Cbx4 sumoylates Prdm16 to regulate adipose tissue thermogenesis. *Cell Reports*.

[13] He L., Tang M., Xiao T. (2018). Obesity-associated miR-199a/214 cluster inhibits adipose browning via PRDM16–PGC-1*α* transcriptional network. *Diabetes*.

[14] Liao J., Jiang J., Jun H. (2018). HDAC3-selective inhibition activates brown and beige fat through PRDM16. *Endocrinology*.

[15] Huang S., Shao G., Liu L. (1998). The PR domain of the Rb-binding zinc finger protein RIZ1 is a protein binding interface and is related to the SET domain functioning in chromatin- mediated gene expression. *The Journal of Biological Chemistry*.

[16] Pinheiro I., Margueron R., Shukeir N. (2012). Prdm3 and Prdm16 are H3K9me1 methyltransferases required for mammalian heterochromatin integrity. *Cell*.

[17] Trayhurn P., Temple N. J., Van Aerde J. (1989). Evidence from immunoblotting studies on uncoupling protein that brown adipose tissue is not present in the domestic pig. *Canadian Journal of Physiology and Pharmacology*.

[18] Berg F., Gustafson U., Andersson L. (2006). The uncoupling protein 1 gene (UCP1) is disrupted in the pig lineage: a genetic explanation for poor thermoregulation in piglets. *PLoS Genetics*.

[19] Lin J., Cao C., Tao C. (2017). Cold adaptation in pigs depends on UCP3 in beige adipocytes. *Journal of Molecular Cell Biology*.

[20] Zheng Q., Lin J., Huang J. (2017). Reconstitution of UCP1 using CRISPR/Cas9 in the white adipose tissue of pigs decreases fat deposition and improves thermogenic capacity. *Proceedings of the National Acadamy of Sciences of the United States of America*.

[21] Hou L., Shi J., Cao L., Xu G., Hu C., Wang C. (2017). Pig has no uncoupling protein 1. *Biochemical and Biophysical Research Communications*.

[22] Soukas A., Socci N. D., Saatkamp B. D., Novelli S., Friedman J. M. (2001). Distinct transcriptional profiles of adipogenesis in vivo and in vitro. *The Journal of Biological Chemistry*.

[23] Seale P., Conroe H. M., Estall J. (2011). Prdm16 determines the thermogenic program of subcutaneous white adipose tissue in mice. *The Journal of Clinical Investigation*.

[24] Nedergaard J., Cannon B. (2018). Brown adipose tissue as a heat-producing thermoeffector. *Handbook of Clinical Neurology*.

[25] Maeda K., Cao H., Kono K. (2005). Adipocyte/macrophage fatty acid binding proteins control integrated metabolic responses in obesity and diabetes. *Cell Metabolism*.

[26] Michalik L., Auwerx J., Berger J. P. (2006). International union of pharmacology. LXI. Peroxisome proliferator-activated receptors. *Pharmacological Reviews*.

[27] Rosen E. D., Sarraf P., Troy A. E. (1999). PPAR gamma is required for the differentiation of adipose tissue in vivo and in vitro. *Molecular Cell*.

[28] Seale P., Bjork B., Yang W. (2008). PRDM16 controls a brown fat/skeletal muscle switch. *Nature*.

